# Nevus Sebaceus of Jadassohn

**Published:** 2015-07-20

**Authors:** Kelly Segars, Jared M. Gopman, Joshua B. Elston, Michael A. Harrington

**Affiliations:** ^a^College of Osteopathic Medicine, Nova Southeastern University, Davie, Fla; ^b^Division of Plastic Surgery, Department of Surgery, Morsani College of Medicine, University of South Florida, Tampa

**Keywords:** nevus sebaceus of Jadassohn, basal cell carcinoma, malignant degeneration, congenital, hamartoma

## DESCRIPTION

**Figure 1:**
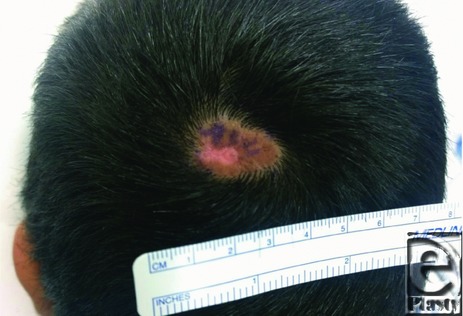
Hairless scalp vertex lesion present since birth that has been slowly enlarging.

**Figure 2:**
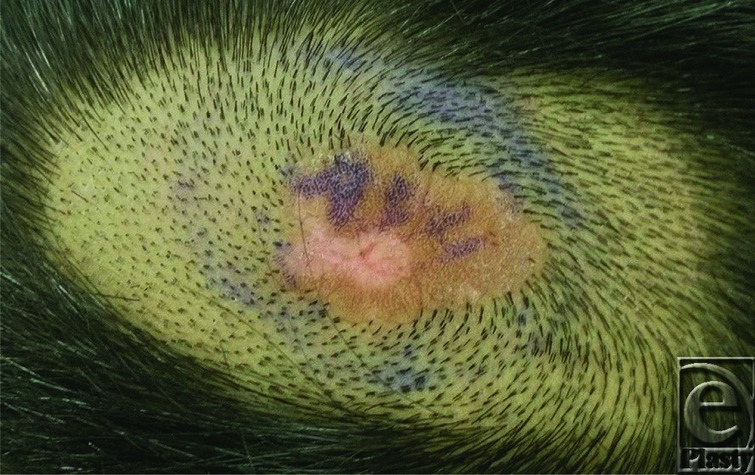
Enlarged view of scalp vertex lesion with a smooth, tan, plaque-like appearance.

A 10-year-old boy presented for evaluation of a slowly growing congenital scalp lesion (Figs 1 and 2). He had no other growth or developmental abnormalities. His mother was concerned about him being teased at school and also that it might be cancerous because it had been growing.

## QUESTIONS

1. **What is nevus sebaceus (NS) of Jadassohn?**
2. **What are the stages of clinical progression?**
3. **What is the rationale for excision versus observation?**
4. **When should excision be performed?**

## DISCUSSION

Nevus sebaceus of Jadassohn is a congenital abnormality first described by the dermatologist Josef Jadassohn in 1895.^1^ It is a congenital hamartomatous lesion with an epithelial and adnexal origin present in approximately 0.3% of newborns that may contain any component of skin including sebaceous and apocrine glands or hair follicles.^2^^-^4 If associated with syndromic features such as mental retardation, central nervous system abnormalities, oculocardiac defects, or skeletal abnormalities, it is called linear nevus sebaceous syndrome, or Schimmelpenning syndrome (a neurocutaneous phakomatosis).

During infancy, NS typically appears as a smooth or velvety yellow-orange well-circumscribed plaque. Classically, it is located on the vertex of the scalp but has been described on other areas such as the face or neck.^4^ At puberty, hormonal changes cause proliferation and hyperplasia of the lesion and lead to a larger and more verrucous appearance that can become quite large.^3^ Later in life, the lesions can develop benign or malignant appendageal tumors that cause further disfigurement.^4^

Individuals with NS may develop benign or malignant tumors, with increasing evidence supporting the former to be the majority. A meta-analysis of 4900 cases found secondary tumor development in 24% of patients, most commonly benign basaloid proliferations such as trichoblastomas.^5^ Malignant transformations have been reported with an average incidence of 8% and ranging from 0% to 22%, most commonly basal cell carcinoma, although there is some controversy of falsely high estimations, given the misdiagnosis of trichoblastomas that appear histologically similar to basal cell carcinoma in earlier reports.^4^^,^^6^^,^^7^

The timing of surgical intervention is controversial. While smaller lesions may be technically easier to excise, younger patients may not be amenable to local anesthesia or able to safely tolerate a general anesthetic. Secondary tumor transformation seems to be seen almost exclusively in adults, as one retrospective analysis found that 96% of all NS-derived malignant tumors occurred in patients older than 18 years and the remaining 4% in patients aged 11 to 17 years.^7^ This has led to the popularized reasoning to excise prior to pubertal enlargement and when local and general anesthesia are well tolerated versus clinically observing until malignant features develop.^4^ Therapeutic management of NS includes full-thickness excision with clear margins. CO_2_ lasers have been shown to enhance cosmesis but can only reach the papillary dermis and are therefore not recommended because of the possibility of malignant degeneration of remaining cells in the lower dermis.^6^

Nevus sebaceus is a rare congenital abnormality that can lead to cosmetic deformity, alopecia, or malignant transformation in rare circumstances. The lesion is usually present at birth, slowly enlarges with age, and typically grows most rapidly during the pubertal hormonal surge. The most common malignant transformation is into basal cell carcinoma, but the most common growth is a benign trichoblastoma. No definitive consensus on surgical intervention versus close observation exists. In the absence of worrisome signs for malignant transformation, management of NS is a decision best made by the child, his or her family, and the surgeon, based on the safety/tolerability of anesthesia, their expectations of oncologic safety, and the understanding of more extensive excision required should increased growth occur.
